# Wells’ Syndrome Associated With Idiopathic Hypereosinophilic Syndrome in a Child: A Case Report

**DOI:** 10.7759/cureus.76349

**Published:** 2024-12-24

**Authors:** Fatmah Altaweel, Adnan Ahmad, Abeer Albazali, Yasmeen Mandani, Alsadat Mosbeh

**Affiliations:** 1 Dermatology, Kuwait Institute for Medical Specializations, Sulibekhat, KWT; 2 Dermatology, Farwaniya Hospital, Kuwait City, KWT; 3 Dermatology and Venereology, Faculty of Medicine, Al-Azhar University, Cairo, EGY

**Keywords:** eosinophilic cellulitis, hypereosinophilia, idiopathic hypereosinophilic syndrome, wells syndrome, wells’ syndrome

## Abstract

Wells' syndrome is a rare inflammatory disease characterized by recurrent, erythematous plaques with histological flame figures, which can be associated with idiopathic hypereosinophilic syndrome (IHES). We present a case of a nine-year-old boy who presented with a one-year history of an itchy rash on his legs associated with peripheral eosinophilia. The rash initially started as an annular plaque and developed raised borders with central hyperpigmentation. A skin biopsy revealed histopathological features consistent with Wells' syndrome, including flame figures and eosinophilic infiltrate. Other investigations revealed hypereosinophilia and bone marrow eosinophilia. Our case demonstrates Wells' syndrome associated with IHES in a child. This overlap renders both diagnosis and treatment challenges. Corticosteroids are effective for Wells' syndrome but may require augmentation with additional therapies for IHES, such as dupilumab. The diagnosis of Wells' syndrome with IHES in this patient underlines the necessity for clinicians to consider and rule out other eosinophilic disorders. It highlights the value of a multidisciplinary strategy to enhance management and optimize outcomes. This case provides valuable insight into the comprehension of rare eosinophilic disorders, but additional research is required in this field to better understand the underlying pathophysiologic mechanisms of these disorders.

## Introduction

The rare inflammatory condition known as Wells' syndrome, or eosinophilic cellulitis, is characterized by recurrent erythematous plaques that look similar to bacterial cellulitis. The presence of "flame figures" is a characteristic histological finding but can be present in other conditions [1]. This condition frequently presents with severe pain and pruritus and can present in different forms, ranging from mild, localized lesions to widespread involvement [1,2]. Although the exact pathophysiology of Wells' syndrome is unknown, it is thought to be linked to an imbalanced immune response in which eosinophils play a key role [2,3]. Eosinophilic infiltration of several organs and persistent eosinophilia are hallmarks of idiopathic hypereosinophilic syndrome (IHES). An absolute eosinophil counts greater than 1.5 x 10⁹/L for more than six months without a known cause is what defines the syndrome [3]. Common signs and symptoms include itchy cutaneous papules, nodules, or plaques; lymphadenopathy; cardiac involvement; and thromboembolic events [2,3]. A range of clinical and histopathological findings are frequently highlighted in studies published in the literature [2,3]. The coexistence of Well’s syndrome and IHES in patients highlights the complexity of eosinophil-mediated diseases and the challenges they pose in their diagnosis and management. We present a case of Wells' syndrome associated with IHES in a child, highlighting the clinical and histopathological features that led us to the diagnosis. Our case report aims to support the existing literature by shedding light on this rare entity in the hope of helping to understand this disease further.

## Case presentation

**Figure 1:**
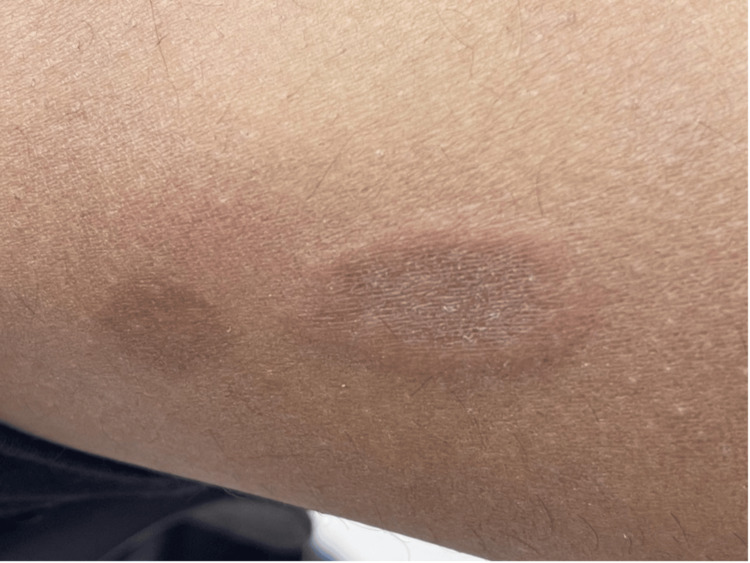
Annular erythematous, non-scaly plaques with indurated raised borders on the right and on the left is a previous lesion that healed with hypermelanosis

**Figure 2:**
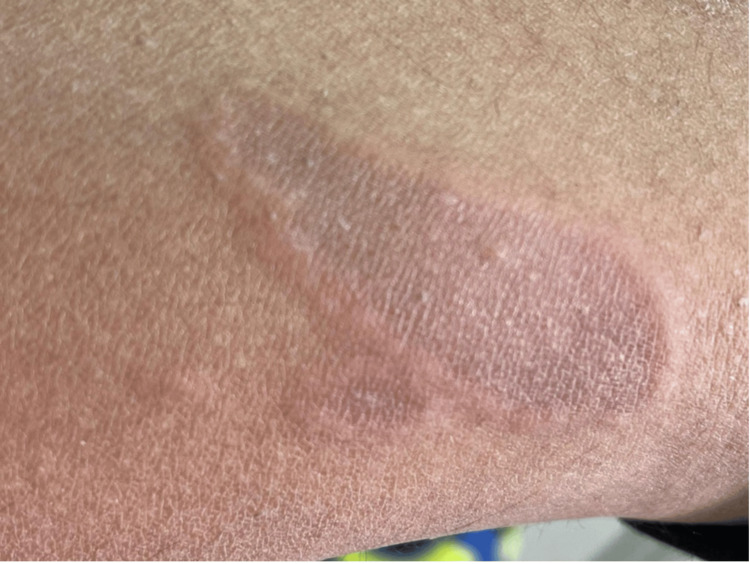
Active lesion showing erythematous plaque, non-scaly, with indurated raised border

**Figure 3:**
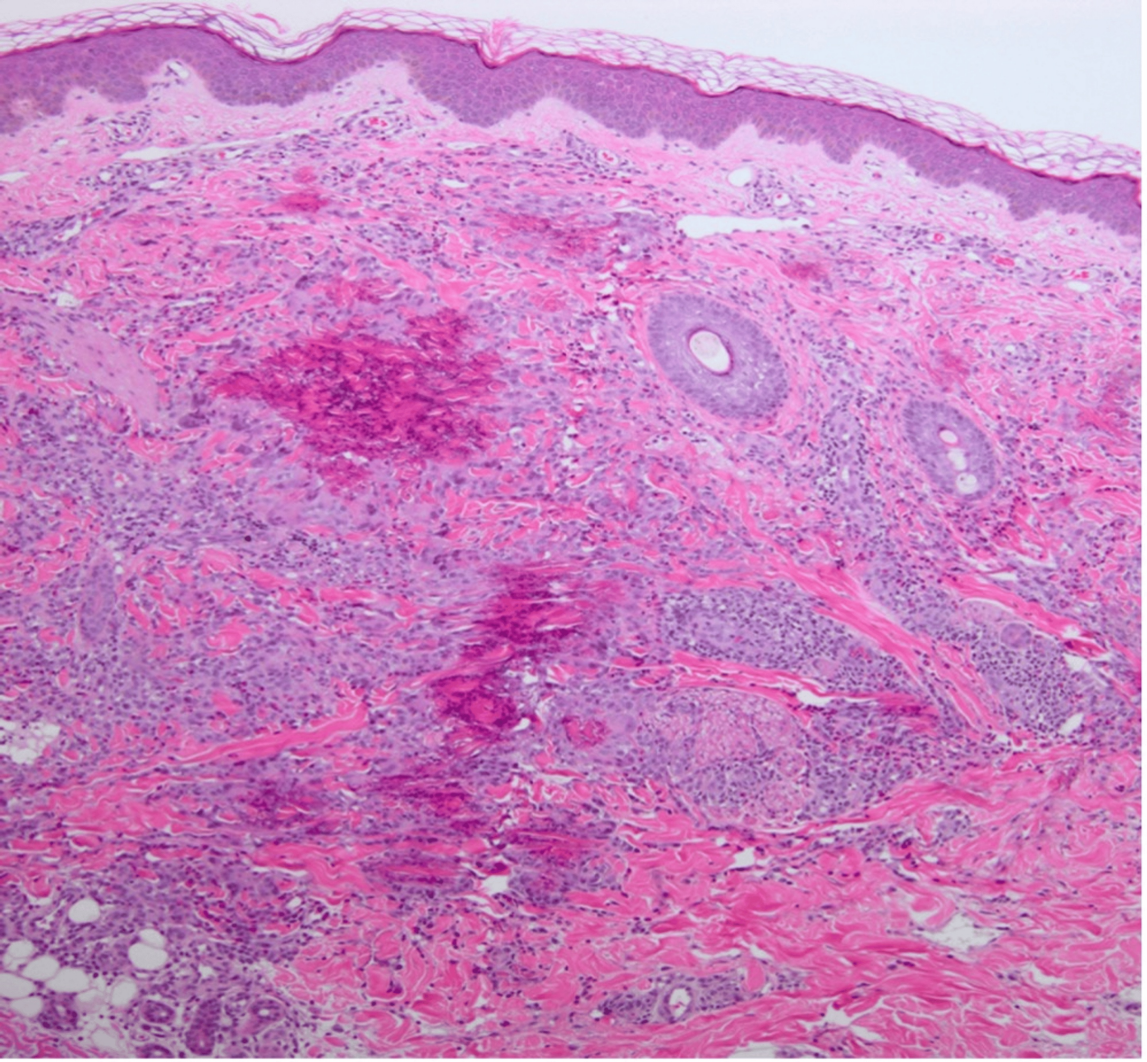
Perivascular and periadnexal inflammatory infiltrate from the skin biopsy

**Figure 4:**
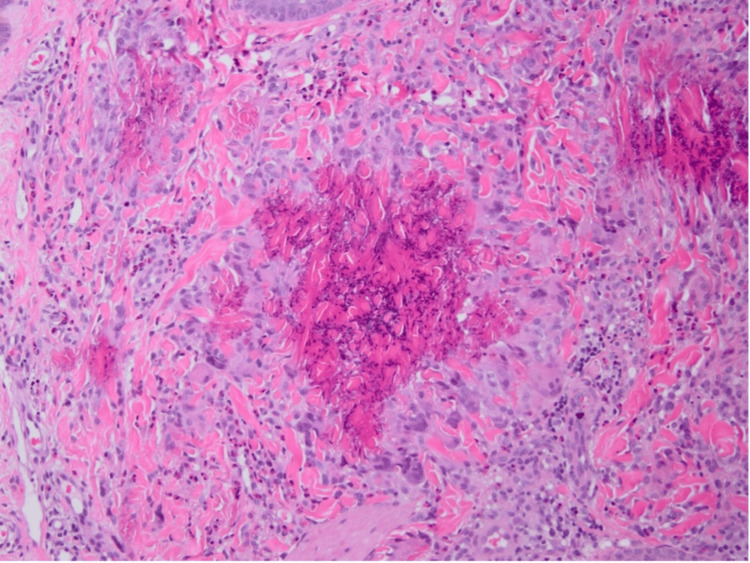
Perivascular and periadnexal inflammatory infiltrates are formed of numerous eosinophils mixed with lymphohistiocytic and giant cells. Flame figures are also noted from the skin biopsy

Herein, we report a case of a nine-year-old boy referred to us from a general pediatrics department due to a persistent itchy skin rash on both legs for one year. Past medical history was notable for lymphadenopathy with peripheral eosinophilia. The patient’s mother reported that the rash initially appeared as a circular lesion on the lower limbs, which later developed a raised border and central spotting. The mother denied recent infections, sick contacts, chronic illnesses, long-term medication intake, or a family history of the same issue. The rash was associated with mild itchiness, which was not relieved by hydrocortisone cream. They denied symptoms such as fever, chills, abdominal pain, nausea, vomiting, diarrhea, chest pain, oral ulcers, palmoplantar changes, recent travel, weight loss, or history of exposure to animals or arthropod bites. Physical examination showed annular erythematous plaques with few scales and indurated raised borders on the bilateral lower limbs (Figures 1-2). Several healed lesions with hyperpigmentation were also noted on the lower limbs. After ruling out fungal infections, a provisional diagnosis of granuloma annulare was made based on the clinical findings. Consequently, a punch biopsy under local anesthesia was performed on an advancing border of a lesion on the medial left lower limb. Histological examination revealed a superficial and deep perivascular, interstitial, and periappendiceal inflammatory infiltrate composed of lymphohistiocytic and giant cells, along with numerous eosinophils and flame figures and normal overlying epidermis (Figures 3-4). Laboratory tests showed hypereosinophilia, with an absolute eosinophil count of 2.1 x 10⁹/L. A recent blood film confirmed reactive hypereosinophilia, and bone marrow aspiration revealed 30% eosinophils but otherwise normal. The erythrocyte sedimentation rate was elevated at 36 mm/hr. Additional tests, including liver, renal, and thyroid function tests, were within normal limits. Serum IgE and tryptase levels, as well as vitamin B12 levels, were all normal. Serum immunoglobulin levels were also within normal limits, and testing for Epstein-Barr virus infection was negative. Serological tests for connective tissue diseases and rheumatoid factor were both negative. Mast cell reactivity was absent, and a pelvi-abdominal ultrasound showed no abnormalities. Genetic testing excluded acute myeloid leukemia, myeloproliferative neoplasms, myelodysplastic syndromes, systemic mastocytosis, and myeloid/lymphoid neoplasms with eosinophilia, as results for PDGFRA, PDGFRB, FGFR1, or PCM1-JAK2 rearrangements were negative. Clonal hematopoiesis mutations and C-Kit mutations were also negative. T-cell rearrangement studies indicated polyclonal results. Therefore, based on the clinical, laboratory, and histological findings, the patient was diagnosed with Wells’ syndrome associated with IHES. The patient was treated with topical hydrocortisone cream 1%, which showed slight improvement after two weeks.

## Discussion

In this case, the patient was diagnosed with Wells' syndrome associated with IHES, highlighting the distinct clinical, histological, and laboratory findings that supported the diagnosis. Due to its similarity to other dermatological conditions such as cellulitis or granuloma annulare, Wells' syndrome, which is characterized by recurrent erythematous plaques and histological evidence of eosinophilic infiltrate with flame figures, frequently poses diagnostic challenges [4,5]. The histological hallmark of Wells’ syndrome, the flame figures, are eosinophil granule proteins deposited on collagen fibers, which were clearly observed in both the skin and lymph node biopsies of our patient [4,5]. IHES is a multisystem disorder characterized by persistent eosinophilia and eosinophilic infiltration of various organs. In this case, the patient’s hypereosinophilia and elevated eosinophil count, coupled with clinical manifestations such as pruritic skin lesions and elevated eosinophils in the bone marrow and peripheral blood, were consistent with the diagnostic criteria for IHES [5]. This syndrome often complicates the clinical picture by affecting multiple systems, including the skin, heart, and gastrointestinal tract. The differential diagnosis for IHES includes primary HES associated with myeloproliferative neoplasms, such as those with genetic mutations in PDGFRA, PDGFRB, FGFR1, and JAK2. These conditions often present with elevated serum tryptase and vitamin B12 levels, hepatosplenomegaly, thrombocytopenia, and anemia. However, none of these features are present in our patient, making primary HES less likely. Additionally, the T-lymphocytic variant of HES, characterized by abnormal T-cell populations detectable through flow cytometry, was overlooked because there were no such anomalies in our case. Familial HES, which typically involves a family history of eosinophilia, was excluded due to the absence of relevant familial patterns [6]. Despite its rarity, the simultaneous presence of both IHES and Wells' syndrome has been reported in the literature. This overlap raises the possibility of a common pathogenic mechanism that may be connected to an abnormal eosinophilic reaction [7]. The literature on such associations is limited, but studies have indicated that the presence of sustained eosinophilia could exacerbate or mimic symptoms of Wells’ syndrome [7]. The underlying mechanism might be a dysregulated eosinophilic response, in which the systemic involvement observed in IHES and the skin manifestations of Wells' syndrome are both caused by excessive eosinophil activation and degranulation [4]. Because of their anti-inflammatory properties and capacity to lower eosinophil counts, corticosteroids are frequently used in the treatment of Wells' syndrome [8]. However, a combination of systemic corticosteroids and other treatments that target eosinophils, like dupilumab, can be helpful where eosinophilia is systemic and frequently more severe [9,10]. The need for clinicians to be aware of rare eosinophilic disorders, the significance of taking into account several overlapping conditions, and the benefits of a multidisciplinary approach in managing complex cases are all highlighted.

## Conclusions

Our case illustrates Wells' syndrome associated with IHES and highlights the challenge clinicians could face when diagnosing and managing eosinophil-associated conditions. The patient's presentation illustrates how complicated these overlapping syndromes can be. Wells' syndrome can be difficult to diagnose because of its nonspecific cutaneous findings and histopathological flame figures, which can present in other conditions. A diligent approach is necessary, as evidenced by the presence of IHES. The fact that both disorders have been identified points to a possible pathogenic mechanism that involves dysregulated eosinophilic responses, but the etiology is unknown according to the literature. The treatment of Wells' syndrome with IHES requires a tailored approach that includes topical and systemic treatments with close observation of eosinophil counts and clinical response. The case highlights the necessity of multidisciplinary management and the consideration of several eosinophilic disorders in the differential diagnosis by clinicians in order to enhance patient outcomes. Further research is required to understand this disorder.
